# Relevance of Indirect Transmission for Wildlife Disease Surveillance

**DOI:** 10.3389/fvets.2016.00110

**Published:** 2016-11-30

**Authors:** Martin Lange, Stephanie Kramer-Schadt, Hans-Hermann Thulke

**Affiliations:** ^1^Department of Ecological Modelling, Helmholtz Centre for Environmental Research Leipzig – UFZ, Leipzig, Germany; ^2^Leibniz Institute for Zoo and Wildlife Research, Berlin, Germany

**Keywords:** indirect transmission, wildlife surveillance, wild boar, FMD, simulation model, contingency planning, environmental transmission, individual-based *R*_0_

## Abstract

Epidemiological models of infectious diseases are essential tools in support of risk assessment, surveillance design, and contingency planning in public and animal health. Direct pathogen transmission from host to host is an essential process of each host–pathogen system and respective epidemiological modeling concepts. It is widely accepted that numerous diseases involve indirect transmission (IT) through pathogens shed by infectious hosts to their environment. However, epidemiological models largely do not represent pathogen persistence outside the host explicitly. We hypothesize that this simplification might bias management-related model predictions for disease agents that can persist outside their host for a certain time span. We adapted an individual-based, spatially explicit epidemiological model that can mimic both transmission processes. One version explicitly simulated indirect pathogen transmission through a contaminated environment. The second version simulated direct host-to-host transmission only. We aligned the model variants by the transmission potential per infectious host (i.e., basic reproductive number *R*_0_) and the spatial transmission kernel of the infection to allow unbiased comparison of predictions. The quantitative model results are provided for the example of surveillance plans for early detection of foot-and-mouth disease in wild boar, a social host. We applied systematic sampling strategies on the serological status of randomly selected host individuals in both models. We compared between the model variants the time to detection and the area affected prior to detection, measures that strongly influence mitigation costs. Moreover, the ideal sampling strategy to detect the infection in a given time frame was compared between both models. We found the simplified, direct transmission model to underestimate necessary sample size by up to one order of magnitude but to overestimate the area put under control measures. Thus, the model predictions underestimated surveillance efforts but overestimated mitigation costs. We discuss parameterization of IT models and related knowledge gaps. We conclude that the explicit incorporation of IT mechanisms in epidemiological modeling may reward by adapting surveillance and mitigation efforts.

## Introduction

Host–pathogen models play an essential role in epidemiology (1). Epidemiological models are widely used to support risk assessment, surveillance design, and contingency planning (2–5). The driving force of any infectious disease is the transmission of the pathogen to susceptible hosts (6, 7), and its adequate representation in epidemiological models is therefore of crucial importance (8, 9).

The relevance of indirect transmission (IT) without a vector or reservoir, but through contaminated environment, was demonstrated for pathogenic viruses, bacteria, prions, and macroparasites. Examples include highly contagious diseases of wildlife and livestock like foot-and-mouth disease [FMD (10), reviewed in Ref. (11, 12)], classical swine fever [CSF; (13, 14)], bovine tuberculosis [bTB; (15, 16)], brucellosis (17), avian influenza [AIV; (18)], porcine reproductive and respiratory syndrome [PRRS; (19)], and chronic wasting disease [CWD; (20)]. Zoonotics and diseases of man with IT mode include infections with influenza viruses (21), cholera bacteria (22, 23), hantaviruses (24), and *Salmonella* bacteria (25). For several pathogens, longevity outside the host was investigated under experimental conditions [see, e.g., Ref. (26) for FMD, CSF, BVDV, and PPV; (27) review FMD; (28) review poultry diseases; (29) review CSF; (30) CSF; (31, 32) AIV; (33) Influenza A, B; (34) cholera].

The necessity to incorporate indirect environmental transmission in epidemiological models was already claimed by several authors (20, 35, 36). Despite this fact, only recent modeling studies considered this transmission mode explicitly [(18) AIV; (20) CWD; (37) cholera; (38) brucellosis]. Instead, the majority of epidemiological models follow a century-old postulate by modeling transmission proportionally to both the current number of infectious and the current number of susceptible individuals (39). Using this approach, Breban (40) elaborated the theory of incorporating IT in epidemiological models. It is not always necessary, indeed, to explicitly model all possible routes of pathogen transmission. One may argue that, for example, infectiousness of environmental contamination being short compared to the host infectious period, and then nothing is lost by summarizing everything in increased estimates of direct transmission (DT) (40). However, if empirical evidence suggests a more fundamental role of pathogen transmission through an environmental pathway, then the previous model paradigm does circumvent the explicit consideration of the biologically independent mechanisms. Such mechanisms may respond differently to interference, e.g., to measures or treatments. Summarizing transmission models, hence, do not allow inferences to be made concerning the role of pathogen stages that can persist outside of their host. Interestingly, studies assessing the impact of IT on disease dynamics or disease mitigation are rare [see, e.g., Ref. (18), for example, Ref. (41–43)].

Explicit consideration of an indirect environmental transmission mode may not only be of serious relevance to understand experimental results or the dynamics of host–pathogen systems [e.g., Ref. (40, 43, 44)]. We claim that the explicit inclusion of environmental transmission in models of wildlife diseases may be necessary for adequate predictions in the context of management activities, e.g., surveillance, mitigation, and contingency planning. Further, IT is particularly relevant in socially organized wildlife species, where direct contact is mainly restricted to the social group, and for multi-host pathogens, where direct contact between species is rare (45, 46). We addressed this hypothesis using a parameterized stochastic spatially explicit, individual-based model (SEIBM) designed for studying infectious diseases in landscape-scale populations of social (47–50) and multi-species wildlife hosts (51, 52).

We used the host–pathogen system of FMD in large wild boar (*Sus scrofa*) populations as a biological example. The wild boar is a social species, widely distributed in many parts of the world. It is the most abundant large mammal species in Europe (53) with increasing geographic range and population densities (53, 54) maintaining a number of infectious diseases (55, 56). FMD is one of the economically most important livestock diseases, which can be devastating in case of an incursion, like in the outbreaks in the UK in 2001 with more than 6.5 million animals culled and economic losses estimated at 5 billion £ (57, 58). FMD affects approximately 70 species of cloven-hoofed domestic and wild animals including wild boar (59). However, epidemiology of FMD in European wildlife populations is largely unknown. The FMD virus (FMDV) can survive outside the host for hours to months, depending on the environmental conditions. In pig slurry, FMDV was detectable for 14 days at 20°C and more than 100 days at 5°C in an experiment by Bøtner and Belsham (26). In a recent outbreak of FMD in wildlife and livestock in Bulgarian Thrace in 2011, wild boars were detected as being virus- and seropositive for FMD, suggesting the potential involvement of the species in FMD epidemics (59, 60).

The objective of this study was to evaluate whether infections with IT may require different surveillance and mitigation efforts than predicted by models based on DT. To this end, we extracted from the SEIBM seroprevalence time series as obtained under surveillance conditions and compared measures important for outbreak mitigation such as time to detection and the minimum sample size needed for disease surveillance.

## Materials and Methods

### Model Description

#### Overview

The FMD wildlife model was based on a spatially explicit, stochastic, individual-based demographic model for wild boars (*S. scrofa*) in a geographic area with suitable habitat. Superimposed is a transmission and disease course model for the FMDV. Epidemiological data on FMDV infections in wild boar are available from the field (59) and laboratory experiments (61, 62). The model is documented following the ODD protocol [Overview, Design, and Details; (63, 64)].

##### Purpose

The aim of the modeling study was to provide an experimental environment to test the hypothesis that neglect of pathogen persistence outside its host is an inappropriate simplification from the perspective of surveillance or contingency planning. The model was designed to compare the predictions between explicit IT and equivalently parameterized DT. For this purpose, two model variants were constructed only differing by the exclusion (DT) or inclusion (IT) of an environmental transmission model. Hence, the following model documentation is representative for all simulations performed with the submodels of direct and IT substituting each other (see Virus Transmission in Section “Details”).

##### State Variables and Scales

The model comprises two major components: spatial habitat units and wild boar individuals. All processes take place on a raster map of spatial habitat units. Each cell represents a functional classification of the landscape denoting habitat quality and a scalar value denoting environmental pathogen load. The cells of the model landscape represent 4 km^2^ (2 km × 2 km), encompassing a boar group’s core home range (65). State variables comprise boar habitat quality of the grid cell. At run time, habitat quality is interpreted as breeding capacity, i.e., the number of female boars that are allowed to have offspring [explicit density regulation; (66)]. Furthermore, an FMDV state of the habitat cell represents environmental virus load and accumulates infection pressure as shed by viremic animals.

State variables of host individuals are the wild boar’s age in weeks [where 1 week represents the approximate FMD infectious period in wild boar; (61, 62)], resulting in age classes: piglet (<8 months ± 6 weeks), sub-adult (<2 years ± 6 weeks), and adult (67). Each host individual has a location, which denotes its home range cell on the raster grid as well as its family group. The individual host animal comprises an epidemiological status (*susceptible, infected*, or *immune* after recovery or due to transient maternal antibodies). Sub-adult wild boar may disperse during the dispersal period (i.e., early summer).

##### Process Overview and Scheduling

**Figure 1:**
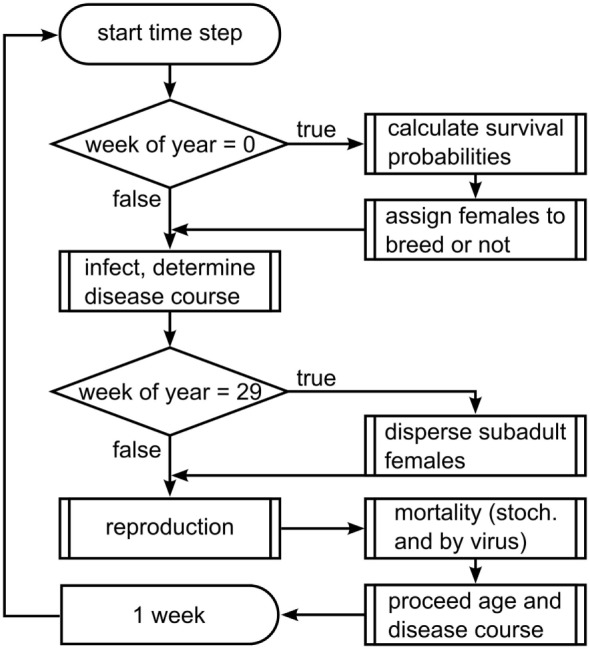
**Flow chart of the scheduling of submodels**.

The model proceeds in weekly time steps and processes are executed in the following order (see Figure 1): virus release, infection, dispersal of subadults, reproduction, death, and aging. In the first week of each year, mortality probabilities are assigned stochastically to represent annual fluctuations in wild boar living conditions, and female wild boars are assigned to breed or not, according to the carrying capacity of their home range cell.

#### Design Concepts

Wild boar population dynamics emerge from individual behavior, defined by age-dependent seasonal reproduction and mortality probabilities and age- and density-dependent dispersal behavior, all including stochasticity. The epidemic course in the DT model emerges from virus transmission within and between groups and wild boar dispersal. The epidemic course in the IT model emerges from virus excretion by infectious hosts, survival dynamics of infectious virus outside the host, contact to infectious doses, and wild boar dispersal.

We included stochasticity by representing demographic, behavioral, and pathogen parameters as probabilities or probability distributions. Annual fluctuations of living conditions are realized by annually varying mortality rates.

#### Details

##### Initialization

The model landscape represents 60 km × 60 km of connected wildlife habitat without barriers. The specified extent ensures that the epidemic wave does not reach the edge of the landscape before detection in any simulation. The 900 grid cells were randomly initialized with integer values of local breeding capacity in range 0, …, 3. Breeding capacity was scaled to result in an average wild boar density of 5 hosts/km^2^ in January, i.e., before the reproductive season (68, 69). The average population size in January was 18,000 individuals.

One boar group was released to each habitat cell, where group size is six times breeding capacity. Initial age distributions were taken from the results of a 100 years model run [see Table S1 in Supplementary Material; (48)].

##### Input

The applied model setup does not include any external inputs or driving variables.

##### Submodels

Submodels are described where essential to understand the study. The Supplementary Material contains the complete descriptions of all submodels. A list of parameters with their values and sources is given in Table S2 in Supplementary Material.

##### Virus Release

The virus was released to the population by infection of five wild boars, randomly selected from the nine most central habitat cells. Release takes place in the sixth year of each simulation (see Simulation Experiments) to allow population dynamics to be established. Introduction was chosen in the season of most likely establishment of the infection according to the increasing population numbers, i.e., at the start of the reproductive season of wild boar.

##### Disease Course

The disease course following infection is modeled for each infected individual. The infectious period of a host *t*_inf_ is 1 week. After the infectious period, hosts achieve lifelong immunity. We assumed minimum case lethality (61, 62).

##### Virus Transmission

###### Direct Transmission

Direct transmission in the model is a stochastic process. Parameters determine the probability of contracting the infection from an infectious group mate $P_{\text{inf}}^{\left(i\right)}$ and the probability of contracting the infection from an infectious animal in a neighboring group $P_{\text{inf}}^{\left(e\right)}$ (3 × 3 neighborhood) during 1 week. For each susceptible animal, the probability of becoming infected accumulates over all infectious animals within the group and in the neighborhood:

(1) $$\Pi_{i}=1-{\left(1-P_{\text{inf}}^{\left(i\right)}\right)}^{I_{i}}{\left(1-P_{\text{inf}}^{\left(e\right)}\right)}^{\Sigma_{j}I_{j}},$$

where *I_i_* is the number of infected individuals in the home group *i* and *I_j_* is the number of infected individuals in wild boar groups of the eight neighboring cells *j* ∈ {1, …, 8}. The model iterates over all individuals and stochastically sets each susceptible individual to infected if a uniformly distributed random number *r* drawn from *U*(0, 1) is smaller than Π_*i*_ of its home cell.

###### Indirect Transmission

We modeled indirect virus transmission *via* excretion of infectious material, decay of infectious material by time in the environment (i.e., outside of host individuals), and contact of hosts to infectious material in the environment. At contact, we modeled the effective infection stochastically with the event probability derived from a standard dose–response relation.

The weekly dynamics of the pathogen pool used in the model are based on parameters available from literature on a daily basis. Temporal evolution of the pathogen pool *C* of each cell is an exponential decay process and the term of pathogen load added to the cell:

(2) $$\frac{\mathrm{dC}}{\mathrm{dt}}=-\lambda C+s,$$

with λ being the decay constant λ = ln(2)/*T*_1/2_, *s* being the pathogen added to the cell per time unit, and *t* being time in weeks. Solve

(3) $$C\left(t\right)=\left(C_{0}-\frac{s}{\lambda }\right)e^{-\lambda t}+\frac{s}{\lambda }.$$

Within one time step, *s* is constant. Thus, the pathogen pool can be calculated analytically as

(4) $$C_{t+1}=\left(C_{t}-\frac{s}{\lambda }\right)e^{-\lambda }+\frac{s}{\lambda }$$

The average available dose for uptake during the weekly time step is

(5) $$\bar{C}=\int_{t}^{t+1}C\left(t\right)\;\mathrm{dt}=\frac{C_{t}\left(1-e^{-\lambda }\right)+s}{\lambda }+\frac{s\left(e^{-\lambda }-1\right)}{\lambda^{2}}.$$

The pathogen source *s* for a cell is determined from the number of infectious hosts in the cell and in neighboring cells. Hosts in infectious state excrete infectious material with constant daily rate (parameter *g*; i.e., 7*g* is the weekly excretion), measured in tissue culture infective dose 50% (TCID_50_) per day. A host animal spends a portion of daytime (parameter *p_t_*) in contact areas, i.e., areas subsequently reached by neighboring animal groups. Accordingly, excreted infectious material is distributed to different cells: *g*(1 − *p_t_*) doses adding to the pool of the home cell of the host, while 1/8*g p_t_* doses are added to each of the eight neighboring cells. Therefore, the pathogen added to a cell on a weekly basis is:

(6) $$s=7g\left(\left(1-p_{t}\right)I_{i}+1∕8\;p_{t}\sum_{j}I_{j}\right).$$

Per host, individual contact to infectious material in the environment is determined as constant share (parameter *u* on a daily basis; i.e., 7*u* corresponds to the weekly share) of the available dose $\bar{C}$ in its home range cell. The weekly contact dose CD is

(7) $$\text{CD}=7u\bar{C}.$$

**Figure 2:**
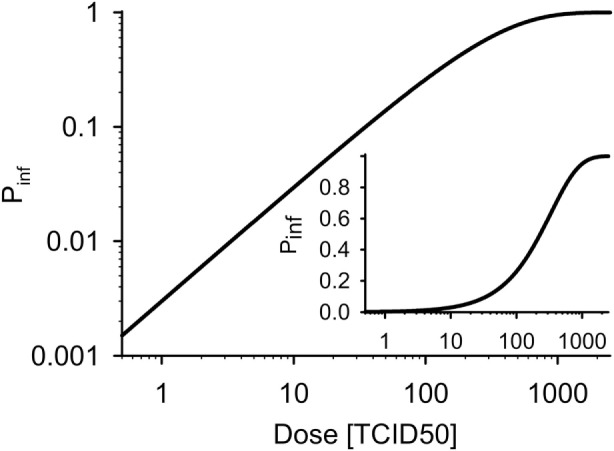
**Dose–response curves for wild boar (*P*_TCID50_ = 0.003)**. Inset: linear ordinate.

Effective infection after contact to a particular dose of infectious material is modeled stochastically as a binomial chance process so that the individual’s weekly probability of becoming infected follows an exponential dose–response relation:

(8) $$P_{\text{CD}}=1-{\left(1-P_{\text{TCID50}}\right)}^{\text{CD}},$$

with *P*_TCID50_ being the probability of infection after contact to one TCID_50_ dose. Figure 2 shows the dose–response curve for *P*_TCID50_ = 0.003 (70, 71).

### Parameters, Simulation Experiments, and Analysis

#### Parameters

A complete list of all parameters with their values and sources is shown in Table S2 in Supplementary Material.

#### Parameterization of Transmission

In the DT model, the transmission is defined by scaling the two parameters $P_{\text{inf}}^{\left(i\right)}$ and $P_{\text{inf}}^{\left(e\right)}$. In the IT model, an analog to *P*_inf_ can be calculated from Eq. 8 and the dose available from one infectious host. To calculate the available dose, Eq. 5 is applied for 1 week after infection (i.e., parameter infectious period) including the excretion into the environment (i.e., *s* > 0) and for infinite time without further excretion. The total available dose over time is

(9) $${\bar{C}}^{\infty }=\int_{0}^{1}C^{+}\left(t\right)\mathrm{dt}+\int_{0}^{\infty }C^{-}\left(t\right)\mathrm{dt},$$

where *C*^+^(*t*) is the pathogen pool with pathogen excretion starting with *C*_0_ = 0 (Eq. 3). *C*^−^(*t*) is the pathogen pool without pathogen excretion for an initial pool equal to the value after the first week [i.e., *C*_0_ = *C*^+^(1)]. Solve

(10) $${\bar{C}}^{\infty }=\frac{s}{\lambda }$$

or, without stressing mathematics, it is the product of added material *s* and average lifetime of the pathogen in the environment τ = 1/λ.

With Eqs 7 and 8, this gives

(11) $$P_{\text{inf}}^{\left(i\right)*}=1-{\left(1-P_{\text{TCID50}}\right)}^{7us_{i}∕\lambda \;}$$

(12) $$P_{\text{inf}}^{\left(e\right)*}=1-{\left(1-P_{\text{TCID50}}\right)}^{7us_{e}∕\lambda \;},$$

with newly added pathogen *s_i_* = 7*g*(1 − *p_t_*) for within-group transmission and *s_e_* = 7*g*(1/8)*p_t_* for between-group transmission.

**Figure 3:**
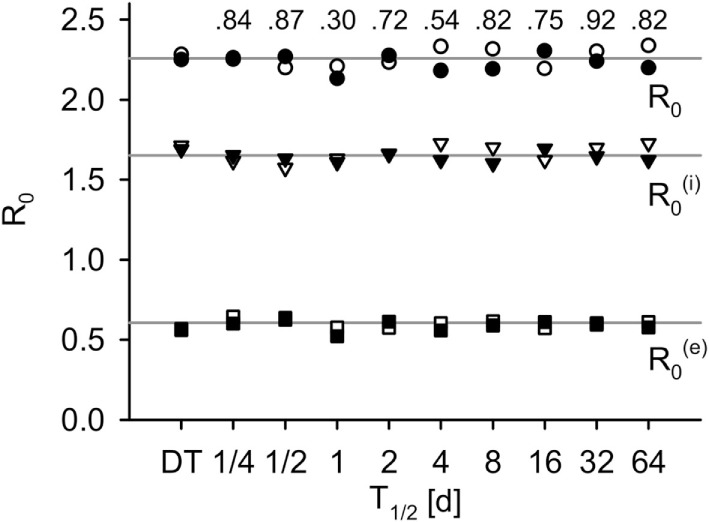
***R*_0_ measured in simulations for the DT model and for the IT model with different pathogen half-life, in total, within-group and between-group component (average of 500 simulations)**. Black: without population dynamics, white: with population dynamics. Lines indicate the theoretical values, $R_{o}^{\left(i\right)}=\text{1.653}$ and $R_{o}^{\left(e\right)}=\text{0.606}$. Numbers indicate *p*-values of two-sided Mann–Whitney *U* tests of total *R*_0_ without population dynamics against DT model (*H*_0_: not different from DT).

By choosing $P_{\text{inf}}^{\left(i\right)}=P_{\text{inf}}^{\left(i\right)*}$ and $P_{\text{inf}}^{\left(e\right)}=P_{\text{inf}}^{\left(e\right)*}$, both models produce the same basic reproductive number *R*_0_ (for validation, see Figure 3).

#### Parallel of *R*_0_ in DT and IT Models

The DT model was parameterized to mimic the IT model in terms of the basic reproduction number *R*_0_. Accounting for transmission within and between groups, *R*_0_ was calculated for both scales of spatial transmission separately. This gives the expected number of infections from one case to its group-mates $R_{0}^{\left(i\right)}$ and to the animals of neighboring groups $R_{0}^{\left(e\right)}$, summing up to $R_{0}=R_{0}^{\left(i\right)}+R_{0}^{\left(e\right)}$.

In the DT model with an infectious period of 1 week, *R*_0_ is a linear function of *P*_inf_:

(13) $$R_{0}^{\left(i\right)}=S_{i}P_{\text{inf}}^{\left(i\right)}$$

(14) $$R_{0}^{\left(e\right)}=S_{e}P_{\text{inf}}^{\left(e\right)}$$

*S_i_* is the number of susceptible hosts in the group of the infectious individual. *S_e_* is the number of susceptible hosts in its neighboring groups.

We can calculate *R*_0_ from the parameters of the IT model using Eqs 11 and 13 for within-group transmission and Eqs 12 and 14 for between-group transmission:

(15) $$R_{0}^{\left(i\right)}=S_{i}\left(1-{\left(1-P_{\text{TCID50}}\right)}^{7us_{i}∕\lambda \;}\right)$$

(16) $$R_{0}^{\left(e\right)}=S_{e}\left(1-{\left(1-P_{\text{TCID50}}\right)}^{7us_{e}∕\lambda \;}\right)$$

**Figure 4:**
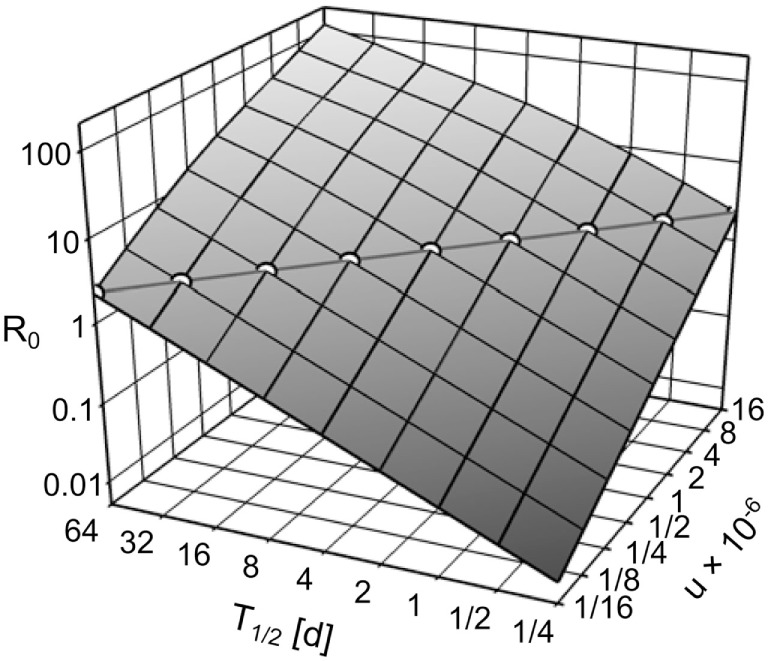
***R*_0_, depending on the environmental pathogen half-life *T*_1/2_ and on the uptake rate *u***. Circles along the diagonal line show the realizations of *T*_1/2_ and *u* used in the simulation experiments (*u* × *T*_1/2_ = 4 × 10^−6^), resulting in *R*_0_ = 2.259.

The exponent in Eqs 15 and 16 can be transformed to 7*us*/λ = 7*usT*_1/2_/ln(2). Thus, *R*_0_ in the IT model can be kept constant over arbitrary pathogen half-life *T*_1/2_ by compensatory scaling of the uptake *u*, i.e., *u* × *T*_1/2_ is constant (see Figure 4). With pathogen half-life approaching 0, the IT model becomes equivalent to the DT model as pathogen uptake becomes instantaneous.

#### Independent Variables

The primary independent variable was the pathogen half-life *T*_1/2_.

#### Simulation Experiments

We performed simulations for the IT model with environmental pathogen half-life *T*_1/2_ ∈ {1/8, 1/4, 1/2, …, 32, 64} days (Figure 4). To keep *R*_0_ constant over all IT simulations, we scaled *u* according to *u* = 4 × 10^−6^/*T*_1/2_. All parameter combinations resulted in *R*_0_ = 2.259. For comparison, we repeated the simulations with the DT model. To achieve the same *R*_0_ as the IT model, transmission parameters were scaled to $P_{\text{inf}}^{\left(i\right)}=0.087$ and $P_{\text{inf}}^{\left(e\right)}=0.00379$. Each parameter set was repeated 500 times.

We performed supplementary simulations to measure an individual-based equivalent of *R*_0_ (20) in order to verify accordance of the transmission model with the theoretical calculations for the basic reproduction number. This was achieved by allowing only the first disease case per model run to be infectious and count of the number of secondary infection in the initially infected cell and in its neighboring cells. The theoretical calculations neglect population turnover, therefore in the third set of simulations, reproduction and mortality were deactivated from the week of pathogen introduction onward. The model runs for 100 times the pathogen half-life after the initial infection to make sure that the environmental reservoir completely decayed and no secondary infections were missed in the analysis.

#### Dependent Variables

We recorded seroprevalence time series for each run on a weekly basis as the first order dependent variable. These prevalence time series were then used to determine second-order dependent variables: (1) time to detection for fixed weekly sample sizes, (2) size of the outbreak at the time of detection, and (3) sample sizes needed to detect the disease within an *a priori* specified time frame. For second-order dependent variables, see Section “Analysis.”

#### Analysis

We mimicked systematic surveillance on the seroprevalence outcome *p* of the DT and the IT model deriving the following second-order dependent variables from prevalence time series.

##### Time to Detection

Given a weekly sample size *n* and seroprevalence *p*, the probability to not find any seropositives in a particular week *t* is

(17) $${\hat{P}}_{0}\left(t\right)={\left(1-p\left(t\right)\right)}^{n}.$$

The probability of not finding any seropositives until the given week can be determined as

(18) $$P_{0}\left(t\right)=\prod_{i=0}^{t}{\hat{P}}_{0}\left(t\right).$$

Hence, the probability to detect the disease until the given week is

(19) $$P_{D}\left(t\right)=1-\prod_{i=0}^{t}{\hat{P}}_{0}\left(t\right).$$

For each model run, the first week of *P_D_*(*t*) ≥ 0.95 determines the time of detection. Subtracting the week of virus incursion, this gives the time to detection *t_D_* of the individual run. The geometric mean of the distribution over the runs gives the time to detection *t_D_* with 95% confidence.

Sample sizes for the underlying surveillance scheme were determined on a monthly basis according to the following equation (72):

(20) $$n_{\text{month}}=\left(1-{\left(1-\text{CL}\right)}^{\frac{1}{N\times p}}\right)\;\left(N-\frac{N\times p-1}{2}\right),$$

with true population size *N*. Parameters of interest were CL = 95%, *p* = 5% and 1%. The required sample size was 58.3 per month (14 per week) for *p* = 5% and 295.6 per month (69 per week) for *p* = 1%.

##### Outbreak Size

The area affected by the disease (area of cells infected) before detection *A_aff_* was determined as a measure of the spatial extent of the outbreak.

##### Required Sample Size

The probability to detect the disease before the given week is calculated according to Eq. 19. This gives the weekly sample size needed to detect the disease in a given time frame *t* for a given seroprevalence time series:

(21) $$n_{D}=\frac{\ln\left(1-\text{CL}\right)}{\ln\prod_{i=0}^{t}\left(1-p\left(i\right)\right)}.$$

We calculated the required weekly sample sizes for each model run.

##### Statistical Analysis

For each simulated value of *T*_1/2_ in the IT model, we compared distributions of time to detection *t_D_* and weekly sample size needed *n_D_* to the outcome of the DT model using the Mann–Whitney *U* test (*H*_0_: distribution with IT not greater than distribution with DT). Similarly, distributions of *A_aff_* were compared to the outcome of the DT model using the Mann–Whitney *U* test (*H*_0_: distribution with IT not less than distribution with DT). Significance was defined as *p*-value < 0.01.

## Results

### Basic Reproduction Number

The individual-based equivalent to *R*_0_ did not differ systematically from the theoretical calculations (compare points to lines in Figure 3). Differences between IT and DT models were not significant (Mann–Whitney *U*, without population dynamics: *p* ≥ 0.3, black fill and numbers in Figure 3; with population dynamics: *p* ≥ 0.35, white fill in Figure 3).

### Seroprevalence

**Figure 5:**
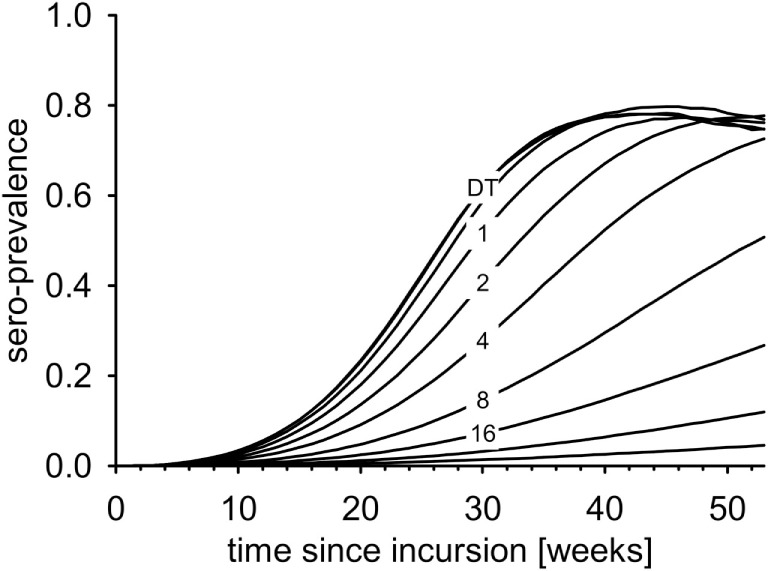
**Average seroprevalence over the first year after virus incursion for the DT model and for the IT model with different pathogen half-life (numbers, in days)**.

Seroprevalence increased most rapidly in the DT model (Figure 5). The first maximum was reached after less than 40 weeks. In the IT model with equal *R*_0_, the increase of seroprevalence slowed down with increasing pathogen half-life (Figure 5, numbers).

### Time to Detection

**Figure 6:**
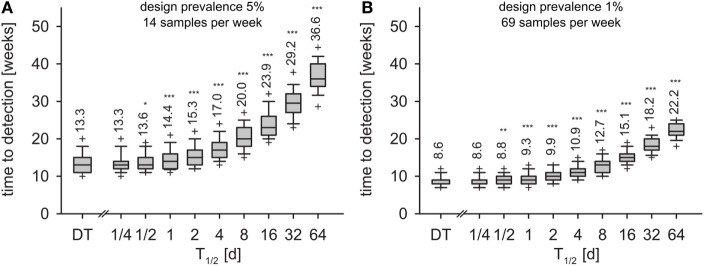
**Time to detection for monthly design prevalence of 5% (A) and 1% (B) for the DT model and for the IT model with different pathogen half-life**. Outlier symbols (+) show 5 and 95% quantiles. Text shows geometric means. Asterisks show significance of Mann–Whitney *U* test against DT model (*H*_0_: not greater than DT; *p < 0.05, **p < 0.01, ***p < 0.001).

In the first experiment, i.e., detection of 5% seroprevalence with 95% confidence within one month of sampling, the surveillance design required 14 samples per week. Applying this sample size to the time series of the DT model, the disease was detected 13.3 weeks after incursion with 95% confidence (geometric mean, Figure 6A, left-most box). With the IT model, time to detection depended on the half-life of pathogen *T*_1/2_ (Figure 6A). Already at *T*_1/2_ > 1 day, detection times were significantly longer than in the DT model (Mann–Whitney *U* test, *p* < 0.01). For half-life of 16 days, time to detection increased to 23.9 weeks. When half-life was 64 days (maximum simulated), time to detection more than doubled compared to the DT model and reached 36.6 weeks.

In the second experiment (detection of 1% seroprevalence with 95% confidence within 1 month of sampling, 69 samples per week), the DT model resulted in detection within 8.6 weeks (Figure 6B, left-most box). Increase of time to detection was significant for *T*_1/2_ > 1 day (Figure 6B). *T*_1/2_ = 16 days resulted in 15.1 weeks and *T*_1/2_ = 64 days in 22.2 weeks to outbreak detection.

### Outbreak Size

**Figure 7:**
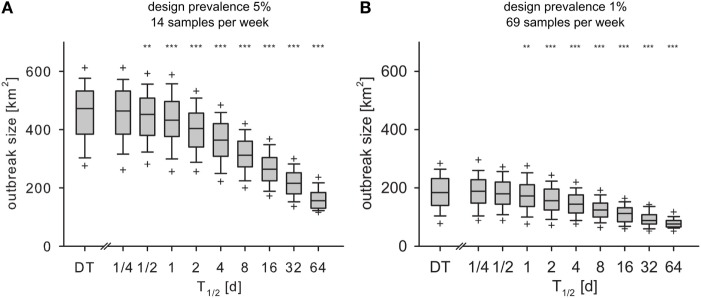
**Outbreak size at the time of detection *A_aff_* for monthly design prevalence of 5% (A) and 1% (B) for the DT model and for the IT model with different pathogen half-life**. Outlier symbols (+) show 5 and 95% quantile. Asterisks show significance of Mann–Whitney *U* test against DT model (*H*_0_: not less than DT; *p < 0.05, **p < 0.01, ***p < 0.001).

In both experiments (design prevalence of 5 and 1%), the spatial extent of the outbreaks *A_aff_* in the IT model decreased significantly compared to the DT model for *T*_1/2_ > 1/2 and *T*_1/2_ > 1 day, respectively (Figures 7A,B).

### Required Sample Size

**Figure 8:**
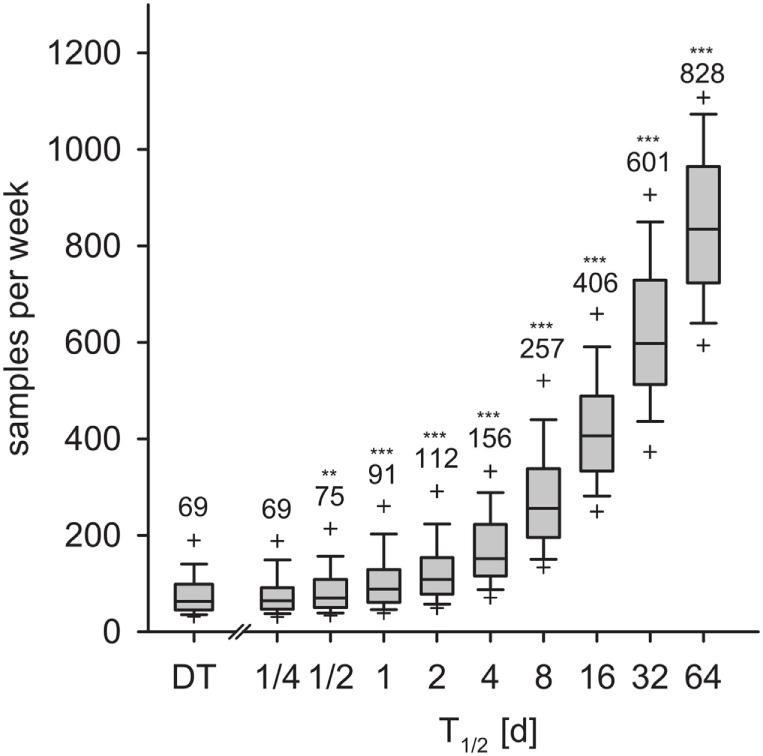
**Required sample size to detect the disease within 9 weeks for the DT model and for the IT model, depending on pathogen half-life**. Outlier symbols (+) show 5 and 95% quantiles. Text shows the average, i.e., the expected sample size for detection with 95% confidence in 9 weeks. Asterisks show significance of Mann–Whitney *U* test against DT model (*H*_0_: not greater than DT; *p < 0.05, **p < 0.01, ***p < 0.001).

We calculated the weekly sample size for detection within 9 weeks with 95% confidence. In the DT model, an average of 69 samples per week was necessary for detection with 95% confidence (Figure 8, left-most box). With the IT model and for pathogen half-life *T*_1/2_ > 1/2 day, the required sample size increased exponentially (Figure 8). With *T*_1/2_ = 16 days, the required sample size was 406 per week. For the maximum half-life of 64 days, 828 samples per week were required for detection within 9 weeks.

## Discussion

For a wildlife host–pathogen system with a social host species, we investigated the consequences of an *a priori* assumption of direct host-to-host transmission in models for surveillance design. We show that the simplified, DT model underestimated necessary sampling efforts by up to one order of magnitude, but overestimated the outbreak area that would receive control or mitigation measures. Thus, simplifying transmission risk as being proportional to the abundance of infectious and susceptible individuals hindered estimation of the most appropriate surveillance and contingency parameters.

The outcomes of a DT model were compared to results from equivalently parameterized IT models with different environmental pathogen persistence. In abstract models, the DT model is a special version of IT assuming persistence time of infectious pathogen in the environment being 0 (40). Here, we are talking about explicit process models tailored to surveillance design in the field. In the field, direct and IT modes correspond with different biological mechanisms that need adequate representation in a model to allow targeted manipulations (see model documentation). The inclusion of environmental transmission is no longer a matter of model re-parameterization but corresponds to a structural change in the model. In this sense both models, the direct and the IT model become fundamentally different. Our results pinpoint the relevance of a decision on whether environmental transmission needs to be represented in a model or not already prior to making predictions. In the logic of our analysis, however, it was necessary to allow seamless transition between models in spite of two alternative transmission mechanisms involved. We have achieved the virtual equivalence of the models while keeping the transmission potential per infected host unchanged.

Environmental transmission in a disease model might be represented assuming prolonged infectiousness of infected hosts along with prolonged half-life of the pathogen in the environment. Logically then, prolonged pathogen persistence in the environment leads to increased transmission potential of the average infected host in turn changing disease dynamics [see, e.g., Ref. (40)]. Here, we were not interested in theoretical variation of the infectious potential of infected hosts across alternative pathogens. Rather, we were addressing alternative models of the same infection, e.g., a pathogen with *R*_0_ established in experiments. This approach was fundamental to the presented comparative assessment of model predictions on a particular disease, i.e., when the DT and the IT version of the model are aligned by the *R*_0_ value.

We focused the comparative assessment of the different transmission models on three measurements for two surveillance schemes: (1) time to detection of an outbreak *t_D_*, (2) spatial extent of the outbreak *A_aff_* at the time of detection, and (3) the sample size required for outbreak detection within a prescribed time frame.

Indirect transmission slowed down the increase in *seroprevalence* compared to DT with equal *R*_0_. An IT route through the environment results in prolonged infectiousness beyond the infectious period of the host. This causes delayed infections compared to the DT mode, where the infectious period of the hosts limits the time span for new infections. Outbreaks governed by IT may progress much slower and hence less obvious.

*Time to detection t_D_* is a central measure to be minimized by a surveillance scheme (73). The underestimated time to detection in the DT model will impede the realized probability of detection of a given surveillance design. Therefore, a surveillance scheme based on the estimates from the DT model [e.g., Ref. (74)] would not meet its aim of detecting an outbreak within the time horizon it was designed for. The pathogen would circulate undetected in the wildlife population longer than expected, therewith increasing the risk of infection of other hosts, e.g., livestock, and the risk of far range spread by transportation or airborne aerosols [e.g., reviewed for FMD in Ref. (75)].

The *spatial extent of the outbreak A_aff_* reflects the area under intervention measures to be implemented after outbreak detection. *A_aff_* was overestimated by the DT model. With IT but equal *R*_0_, the disease spread slower than with DT and also has more time to spread due to later detection. Due to the continuous surveillance scheme with accumulation of chance of detection over time, the longer period of undetected pathogen circulation could not completely compensate the slower spread, thus outbreak size at detection was smaller. Control and restriction zones would be oversized if designed on estimates of undetected spread from a DT model. Thereby, the applied measures would be overly expensive and an unnecessary burden for the livestock sector (76).

The DT model underestimated the *required sample size* per time unit for disease detection within a given time frame. This measure quantifies the effort that is actually necessary to achieve the original aim of the surveillance program, namely, outbreak detection within a prescribed time horizon with given confidence. The extreme increase of the sample size for long pathogen persistence suggests that other methods than testing host individuals for seropositivity may be necessary for the surveillance of certain diseases (77, 78).

Remarkably, time to detection and required sample size differed from the predictions of the DT model for pathogen half-life as short as 1 day. This time span is by almost one order of magnitude shorter than the infectious period of 1 week. This fact emphasizes the relevance of IT, even in absence of extreme pathogen longevity.

The model used in this study has been previously applied for risk assessment (47), for assessment of disease control measures (79), and to contribute to the understanding of wildlife host–pathogen systems (48, 49, 51). In this study, we extend our previous work by the integration of IT and compared surveillance-related predictions of different model versions.

We restricted the model versions to either DT or IT, but did not combine both. Although DT is likely to play a role in most host–pathogen systems with IT mode, we were interested in the differences between the two modes. As the IT model with short pathogen half-life resembles the DT model, we nonetheless examined a continuous transition between the aggregation to DT and the explicit IT model.

Numerous empirical and modeling studies dealt with the quantification of indirect, particularly airborne transmission of FMD and other diseases between domestic livestock holdings [e.g., Ref. (71, 80–84), reviewed for FMD in Ref. (85)], but IT of FMDV in wildlife animals has, to our knowledge, not yet been quantified. We developed a modeling approach that breaks down IT into components that are accessible to experimental measurements, namely pathogen shedding, survival/decay in the environment, contact with infectious material, and infection according to a dose–response relationship. Although some experiments quantified pathogen excretion and secretion of FMDV [reviewed in Ref. (12)] and other pathogens [see, e.g., Ref. (86) for CSF] by domestic animals, knowledge for wildlife is rare (87). The large differences between domestic animal species regarding the shedding rates of FMDV (12, 75) call for further attention to this issue. The same applies for the susceptibility of different species, i.e., the dose–response relation (12, 75). Some quantification for domestic animals can be found in the literature [e.g., Ref. (70, 88) for FMD], but the qualitative relation between dose in the environment and probability of infection is often unclear (89, 90). Survival outside the host has been investigated for several pathogens in animal products and excrements under laboratory conditions (for references, see INTRODUCTION), but further research is necessary for environmental factors that influence pathogen survival. The contact of animals with viral contamination in the environment remains the most uncertain parameter. Here, an inverse parameter fitting approach could aid the quantification. Given assumptions for the other parameters, contact to viral contamination could be estimated from the probability of infection.

Experimental investigations of virus survival outside the host depict striking dependence on temperature and humidity [see, e.g., Ref. (26) for FMD, CSF, BVDV, and PPV; (91) for PRRS virus; (92) for influenza A]. This fact gives rise to seasonal fluctuations of the magnitude of IT. Indeed, for several viral diseases, fluctuations of their transmission were associated with climatic seasonality, partly related to virus survival outside the host [see, e.g., Ref. (92, 93) for influenza viruses; (94) for hepatitis A]. Therefore, climatic factors are expected to play a role in regional variations of the epidemiology of infectious diseases with an IT mode.

With this work, we contribute to the research on IT, which is still in an early stage but attracting increasing attention. Previous work focused on the impact of IT on key figures of host–pathogen systems such as the basic reproductive number (20), disease persistence (41), and formal conditions of relevance for modeling (40).

Our results resemble findings by Wearing et al. (1) and Almberg et al. (20), which show that a neglect of prolonged infectiousness, e.g., through environmental pathogen stages or inappropriate assumptions about the infectious period, may result in an underestimate of *R*_0_, if derived from the prevalence growth rate. Reciprocal, in our study prevalence growth rates decreased under IT despite equal reproductive potential (*R*_0_). Thus, we transferred the findings regarding the relevance of IT from a theoretical underestimation of infection dynamics, i.e., *R*_0_, to the application-oriented context of designing surveillance of any particular wildlife disease, i.e., *R*_0_ being fixed.

We conclude that a simplified aggregation of transmission processes, particularly a neglect of environmental pathogen stages, may considerably bias model predictions of the performance of disease surveillance and mitigation strategies. We state that this applies even for pathogens with an average environmental survival time that is comparatively short compared to the infectious period of the host.

## Author Contributions

ML and H-HT conceived and designed the experiments; ML performed the experiments and analyzed the data; and ML, SK-S, and H-HT developed the model and wrote the manuscript.

## Conflict of Interest Statement

The authors declare that the research was conducted in the absence of any commercial or financial relationships that could be construed as a potential conflict of interest.

## References

[1] Wearing HJ Rohani P Keeling MJ. Appropriate models for the management of infectious diseases. PLoS Med (2005) 2(7):e174. 10.1371/journal.pmed.0020174 16013892 PMC1181873

[2] Thulke HH Application of recent approaches in modelling for animal health. Prev Vet Med (2011) 99(1):1–3. 10.1016/j.prevetmed.2011.01.007 21329994

[3] Bolzoni L Real L De Leo G. Transmission heterogeneity and control strategies for infectious disease emergence. PLoS One (2007) 2(8):e747. 10.1371/journal.pone.0000747 17712403 PMC1945090

[4] Elderd BD Dukic VM Dwyer G. Uncertainty in predictions of disease spread and public health responses to bioterrorism and emerging diseases. Proc Natl Acad Sci U S A (2006) 103(42):15693–7. 10.1073/pnas.0600816103 17030819 PMC1592533

[5] Pineda-Krch M O’Brien JM Thunes C Carpenter TE. Potential impact of introduction of foot-and-mouth disease from wild pigs into commercial livestock premises in California. Am J Vet Res (2010) 71(1):82–8. 10.2460/ajvr.71.1.82 20043786

[6] Thulke HH Selhorst T Müller T. Pseudorabies virus infections in wild boar: data visualisation as an aid to understanding disease dynamics. Prev Vet Med (2005) 68:35–48. 10.1016/j.prevetmed.2005.01.002 15795014

[7] Begon M Bennett M Bowers RG French NP Hazel SM Turner J A clarification of transmission terms in host-microparasite models: numbers, densities and areas. Epidemiol Infect (2002) 129(1):147–53. 10.1017/S0950268802007148 12211582 PMC2869860

[8] Kershenbaum A Stone L Ostfeld RS Blaustein L. Modelling transmission of vector-borne pathogens shows complex dynamics when vector feeding sites are limited. PLoS One (2012) 7(5):e36730. 10.1371/journal.pone.0036730 22590597 PMC3348133

[9] McCallum H Barlow N Hone J. How should pathogen transmission be modelled? Trends Ecol Evol (2001) 16(6):295–300. 10.1016/S0169-5347(01)02144-9 11369107

[10] Eblé PL de Koeijer A Bouma A Stegeman A Dekker A. Quantification of within- and between-pen transmission of foot-and-mouth disease virus in pigs. Vet Res (2006) 37:647–54. 10.1051/vetres:2006026 16777036

[11] Donaldson AI Alexandersen S Sørensen JH Mikkelsen T Relative risks of the uncontrollable (airborne) spread of FMD by different species. Vet Rec (2001) 148:602–4. 10.1136/vr.148.19.602 11386448

[12] Alexandersen S Zhang Z Donaldson AI Garland AJ. The pathogenesis and diagnosis of foot-and-mouth disease. J Comp Pathol (2003) 129(1):1–36. 10.1016/S0021-9975(03)00041-0 12859905

[13] Dewulf J Laevens H Mintiens K De Kruif A Koenen F Airborne transmission of classical swine fever virus under experimental conditions. Vet Rec (2000) 147(26):735–8. 10.1136/vr.147.26.735 11195166

[14] Ribbens S Dewulf J Koenen F Maes D De Kruif A. Evidence of indirect transmission of classical swine fever virus through contacts with people. Vet Rec (2007) 160:687–90. 10.1136/vr.160.20.687 17513834

[15] Hutchings MR Harris S. Quantifying the risks of TB infection to cattle posed by badger excreta. Epidemiol Infect (1999) 122(1):167–73. 10.1017/S0950268898001897 10098801 PMC2809603

[16] Palmer MV Waters WR Whipple DL. Shared feed as a means of deer-to-deer transmission of *Mycobacterium bovis*. J Wildl Dis (2004) 40(1):87–91. 10.7589/0090-3558-40.1.87 15137492

[17] Aune K Rhyan JC Russel R Roffe TJ Corso B Environmental persistence of *Brucella abortus* in the Greater Yellowstone Area. J Wildl Manage (2011) 76(2):253–61. 10.1002/jwmg.274

[18] Rohani P Breban R Stallknecht DE Drake JM. Environmental transmission of low pathogenicity avian influenza viruses and its implications for pathogen invasion. Proc Natl Acad Sci U S A (2009) 106(25):10365–9. 10.1073/pnas.0809026106 19497868 PMC2690603

[19] Brockmeier SL Lager KM. Experimental airborne transmission of porcine reproductive and respiratory syndrome virus and *Bordetella bronchiseptica*. Vet Microbiol (2002) 89(4):267–75. 10.1016/S0378-1135(02)00204-3 12383636

[20] Almberg ES Cross PC Johnson CJ Helsey DM Richards BJ. Modeling routes of chronic wasting disease transmission: environmental prion persistence promotes deer population decline and extinction. PLoS One (2011) 6(5):e19896. 10.1371/journal.pone.0019896 21603638 PMC3094393

[21] Buxton Bridges C Kuehnert MJ Hall CB. Transmission of influenza: implications for control in health care settings. Clin Infect Dis (2003) 37(8):1094–101. 10.1086/378292 14523774

[22] Colwell RR Huq A Environmental reservoir of *Vibrio cholerae*. The causative agent of cholera. Ann N Y Acad Sci (1994) 740(1):44–54. 10.1111/j.1749-6632.1994.tb19852.x 7840478

[23] Kaper JB Morris JG Jr Levine MM. Cholera. Clin Microbiol Rev (1995) 8(1):48–86. 7704895 10.1128/cmr.8.1.48 PMC172849

[24] Kallio ER Klingström J Gustafsson E Manni T Vaheri A Henttonen H Prolonged survival of Puumala hantavirus outside the host: evidence for indirect transmission via the environment. J Gen Virol (2006) 87:2127–34. 10.1099/vir.0.81643-0 16847107

[25] Österberg J Sternberg Lewerin S Wallgren P. Direct and indirect transmission of four *Salmonella enterica* serotypes in pigs. Acta Vet Scand (2010) 52:30. 10.1186/1751-0147-52-30 20459711 PMC2882913

[26] Bøtner A Belsham GJ. Virus survival in slurry: analysis of the stability of foot-and-mouth disease, classical swine fever, bovine viral diarrhoea and swine influenza viruses. Vet Microbiol (2012) 157(1–2):41–9. 10.1016/j.vetmic.2011.12.010 22226541

[27] Bartley LM Donnelly CA Anderson RM Review of foot-and-mouth disease virus survival in animal excretions and on fomites. Vet Rec (2002) 151:667–9. 10.1136/vr.151.22.667 12498410

[28] Soos C Padilla L Iglesias A Gottdenker N Cruz B don M Comparison of pathogens in broiler and backyard chikens on the Gal Pagos Islands: implications for transmission to wildlife. Auk (2008) 125(2):445–55. 10.1525/auk.2008.06235

[29] Edwards S. Survival and inactivation of classical swine fever virus. Vet Microbiol (2000) 73:175–81. 10.1016/S0378-1135(00)00138-3 10785326

[30] Weesendorp E Stegeman A Loeffen WLA. Survival of classical swine fever virus at various temperatures in faeces and urine derived from experimentally infected pigs. Vet Microbiol (2008) 132(3–4):249–59. 10.1016/j.vetmic.2008.05.020 18602226

[31] Tiwari A Patnayak DP Chander Y Parsad M Goyal SM. Survival of two avian respiratory viruses on porous and nonporous surfaces. Avian Dis (2006) 50:284–7. 10.1637/7453-101205R.1 16863083

[32] Brown JD Swayne DE Cooper RJ Burns RE Stallknecht DE. Persistence of H5 and H7 avian influenza viruses in water. Avian Dis (2007) 50:285–9. 10.1637/7636-042806R.1 17494568

[33] Bean B Moore M Sterner B Peterson LR Gerding DN Balfour HH Jr. Survival of influenza viruses on environmental surfaces. J Infect Dis (1982) 146(1):47–51. 10.1093/infdis/146.1.47 6282993

[34] Xu HS Roberts N Singleton FL Attwell RW Grimes DJ Colwell RR. Survival and viability of nonculturable *Escherichia coli* and *Vibrio cholerae* in the estuarine and marine environment. Microb Ecol (1982) 8:313–23. 10.1007/BF02010671 24226049

[35] Caley P Marion G Hutchings MR Assessment of transmission rates and routes, and the implications for management. In: Delahay RJ Smith GC Hutchings MR, editors. Management of Disease in Wild Mammals. Tokyo, Berlin, Heidelberg, New York: Springer (2009). p. 31–52.

[36] Marcé C Ezanno P Weber MF Seegers H Pfeiffer DU Fourichon C. Invited review: modeling within-herd transmission of *Mycobacterium avium* subspecies paratuberculosis in dairy cattle: a review. J Dairy Sci (2010) 93(10):4455–70. 10.3168/jds.2010-3139 20854979

[37] Joh RI Wang H Weiss H Weitz JS Dynamics of indirectly transmitted infectious diseases with immunological threshold. Bull Math Biol (2008) 71(4):845–62. 10.1007/s11538-008-9384-4 19096894

[38] Aïnseba B Benosman C Magal P. A model for ovine brucellosis incorporating direct and indirect transmission. J Biol Dyn (2010) 4(1):2–11. 10.1080/17513750903171688 22881067

[39] Eisenberg JNS Brookhart MA Rice G Brown M Colford JM Jr. Disease transmission models for public health decision making: analysis of epidemic and endemic conditions caused by waterborne pathogens. Environ Health Perspect (2002) 110(8):783–90. 10.1289/ehp.02110783 12153759 PMC1240949

[40] Breban R. Role of environmental persistence in pathogen transmission: a mathematical modeling approach. J Math Biol (2013) 66:535–46. 10.1007/s00285-012-0520-2 22382994 PMC7079992

[41] Sauvage F Langlais M Yoccoz NG Pontier D Modelling hantavirus in fluctuating populations of bank voles: the role of indirect transmission on virus persistence. J Anim Ecol (2003) 72(1):1–13. 10.1046/j.1365-2656.2003.00675.x

[42] Ivanek R Lahodny GE From the bench to modeling – R_0_ at the interface between empirical and theoretical approaches in epidemiology of environmentally-transmitted infectious diseases. Prev Vet Med (2015) 118(2–3):196–206. 10.1016/j.prevetmed.2014.11.003 25441048

[43] Fuller E Elderd BD Dwyer G. Pathogen persistence in the environment and insect-baculovirus interactions: disease-density thresholds, epidemic burnout, and insect outbreaks. Am Nat (2012) 179(3):E70–96. 10.1086/664488 22322229 PMC3814039

[44] De Jong MCM Hagenaars TJ Eble P Transmission of FMDV within and between species: quantification and comparisons. Proceedings of ISVEE Maastricht Wageningen: Wageningen Academic Publishers (2012). 47 p.

[45] Woolhouse MEJ Taylor LH Haydon DT. Population biology of multihost pathogens. Science (2001) 292:1109–12. 10.1126/science.1059026 11352066

[46] Pedersen AB Altizer S Poss M Cunningham AA Nunn CL. Patterns of host specificity and transmission among parasites of wild primates. Int J Parasitol (2005) 35:647–57. 10.1016/j.ijpara.2005.01.005 15862578

[47] Fernández N Kramer-Schadt S Thulke HH Viability and risk assessment in species restoration: planning reintroductions for the wild boar, a potential disease reservoir. Ecol Soc (2006) 11(1):6 10.5751/ES-01560-110106

[48] Kramer-Schadt S Fernández N Eisinger D Grimm V Thulke HH Individual variations in infectiousness explain long-term disease persistence in wildlife populations. Oikos (2009) 118:199–208. 10.1111/j.1600-0706.2008.16582.x

[49] Lange M Kramer-Schadt S Blome S Beer M Thulke HH Disease severity declines over time after a wild boar population has been affected by classical swine fever – legend or actual epidemiological process? Prev Vet Med (2012) 106:185–95. 10.1016/j.prevetmed.2012.01.024 22361000

[50] Alban L Andersen MM Asferg T Boklund A Fernández N Goldbach SG Classical Swine Fever and Wild Boar in Denmark: A Risk Analysis. Copenhagen: Danish Institute for Food and Veterinary Research (2005). 118 p.

[51] Lange M Spatial spread and maintenance of foot-and-mouth disease virus infections in wildlife populations of Thrace region applying epidemiological modelling. Scientific report submitted to EFSA. EFSA Supporting Publications. EFSA J (2012) 9(4):EN–264, 28. 10.2903/sp.efsa.2012.EN-264

[52] Dhollander S Depner K Belsham GJ Salman M Willgert K Thulke HH Evaluating the potential spread and maintenance of foot-and-mouth disease virus in wildlife; general principles and application to a specific scenario in Thrace. Transbound Emerg Dis (2016) 63(2):165–174. 10.1111/tbed.12240 24903641

[53] Artois M Depner KR Guberti V Hars J Rossi S Rutili D. Classical swine fever (hog cholera) in wild boar in Europe. Rev Sci Tech (2002) 21(2):287–303. 10.20506/rst.21.2.1332 11974616

[54] Saez-Royuela DC Telleria JL The increased population of the wild boar (*Sus scrofa*) in Europe. Mamm Rev (1986) 16(2):97–101. 10.1111/j.1365-2907.1986.tb00027.x

[55] Martin C Pastoret PP Brochier B Humblet MF Saegerman C. A survey of the transmission of infectious diseases/infections between wild and domestic ungulates in Europe. Vet Res (2011) 42:70. 10.1186/1297-9716-42-70 21635726 PMC3152899

[56] Ruiz-Fons F Segalés J Gortázar C. A review of viral diseases of the European wild boar: effects of population dynamics and reservoir rôle. Vet J (2008) 176(2):158–69. 10.1016/j.tvjl.2007.02.017 17420149 PMC7110567

[57] Thompson D Muriel P Russell D Osborne P Bromley A Rowland M Economic costs of the foot and mouth disease outbreak in the United Kingdom in 2001. Rev Sci Tech (2002) 21(3):675–87. 10.20506/rst.21.3.1353 12523706

[58] Haydon DT Kao RR Kitching RP The UK foot-and-mouth disease outbreak – the aftermath. Nat Rev Microbiol (2004) 2:675–81. 10.1038/nrmicro960 15263902

[59] EFSA AHAW Panel. Scientific opinion on foot and mouth disease in Thrace. EFSA J (2012) 10(4):2635 10.2903/j.efsa.2012.2635

[60] Alexandrov T Stefanov D Kamenov P Miteva A Khomenko S Sumption KJ Surveillance of foot-and-mouth disease (FMD) in susceptible wildlife and domestic ungulates in Southeast of Bulgaria following a FMD case in wild boar. Vet Microbiol (2013) 166(1–2):84–90. 10.1016/j.vetmic.2013.05.016 23830685

[61] Breithaupt A Depner K Haas B Axandrov T Polihronova L Georgiev G Experimental infection of wild boar and domestic pigs with a foot and mouth disease virus strain detected in the southeast of Bulgaria at the end of 2010. Vet Microbiol (2012) 159(1–2):33–9. 10.1016/j.vetmic.2012.03.021 22503391

[62] Mohamed F Swafford S Petrowski H Bracht A Schmit B Fabian A Foot-and-mouth disease in feral swine: susceptibility and transmission. Transbound Emerg Dis (2011) 58(4):358–71. 10.1111/j.1865-1682.2011.01213.x 21418546

[63] Grimm V Berger U Bastiansen F Eliassen S Ginot V Giske J A standard protocol for describing individual-based and agent-based models. Ecol Modell (2006) 192:115–26. 10.1016/j.ecolmodel.2006.04.023

[64] Grimm V Berger U DeAngelis DL Polhill JG Giske J Railsback SF The ODD protocol: a review and first update. Ecol Modell (2010) 221(23):2760–8. 10.1016/j.ecolmodel.2010.08.019

[65] Leaper R Massei G Gorman ML Aspinall R The feasibility of reintroducing wild boar (*Sus scrofa*) to Scotland. Mamm Rev (1999) 29(4):239–58. 10.1046/j.1365-2907.1999.2940239.x

[66] Jedrzejewska B Jedrzejewski W Bunevich AN Milkowski L Krasinski ZA Factors shaping population densities and increase rates of ungulates in Bialowieza Primeval Forest (Poland and Belarus) in the 19th and 20th centuries. Acta Theriol (1997) 42(4):399–451. 10.4098/AT.arch.97-39

[67] Keuling O Stier N Roth M Annual and seasonal space use of different age classes of female wild boar *Sus scrofa* L. Eur J Wildl Res (2008) 54(3):403–12. 10.1007/s10344-007-0157-4

[68] Sodeikat G Pohlmeyer K Escape movements of family groups of wild boar *Sus scrofa* influenced by drive hunts in Lower Saxony, Germany. Wildl Biol (2003) 9(Suppl 1):43–9.

[69] EFSA Panel on Animal Health and Welfare. Control and eradication of Classic Swine Fever in wild boar. EFSA J (2009) 7(1):932, 199 10.2903/j.efsa.2009.932

[70] French NP Kelly L Jones R Clancy D. Dose-response relationships for foot and mouth disease in cattle and sheep. Epidemiol Infect (2002) 128:325–32. 10.1017/S0950268801006446 12002551 PMC2869826

[71] Garner MG Hess GD Yang X An integrated modelling approach to assess the risk of wind-borne spread of foot-and-mouth disease virus from infected premises. Environ Model Assess (2006) 11:195–207. 10.1007/s10666-005-9023-5

[72] Cannon RM Sense and sensitivity – designing surveys based on an imperfect test. Prev Vet Med (2001) 49(3–4):141–63. 10.1016/S0167-5877(01)00184-2 11311950

[73] Dürr S Zu Dohna H Di Labio E Carpenter TE Doherr MG. Evaluation of control and surveillance strategies for classical swine fever using a simulation model. Prev Vet Med (2013) 108(1):73–84. 10.1016/j.prevetmed.2012.07.006 22858424

[74] Hone J Pech RP Yipo P. Estimation of the dynamics and rate of transmission of classical swine fever (hog cholera) in wild pigs. Epidemiol Infect (1992) 108:377–86. 10.1017/S0950268800049840 1582476 PMC2271981

[75] Kitching RP Hutber AM Thrusfield MV. A review of foot-and-mouth disease with special consideration for the clinical and epidemiological factors relevant to predictive modelling of the disease. Vet J (2005) 169:197–209. 10.1016/j.tvjl.2004.06.001 15727911

[76] Morris RS Sanson RL Stern MW Stevenson M Wilesmith JW. Decision-support tools for foot and mouth disease control. Rev Sci Tech (2002) 21(3):557–67. 10.20506/rst.21.3.1363 12523696

[77] Mouchantat S Haas B Böhle W Globig A Lange E Mettenleiter TC Proof of principle: non-invasive sampling for early detection of foot-and-mouth disease virus infection in wild boar using a rope-in-a-bait sampling technique. Vet Microbiol (2014) 172(1–2):329–33. 10.1016/j.vetmic.2014.05.021 24930983

[78] de Carvalho Ferreira HC Weesendorp E Quak S Stegeman JA Loeffen WLA. Suitability of faeces and tissue samples as a basis for non-invasive sampling for African swine fever in wild boar. Vet Microbiol (2014) 172(3–4):449–54. 10.1016/j.vetmic.2014.06.016 25017975

[79] Lange M Kramer-Schadt S Thulke HH. Efficiency of spatio-temporal vaccination regimes in wildlife populations under different viral constraints. Vet Res (2012) 43(1):37. 10.1186/1297-9716-43-37 22530786 PMC3384476

[80] Donaldson AI Alexandersen S Predicting the spread of foot and mouth disease by airborne virus. Rev Sci Tech (2002) 21(3):569–75. 10.20506/rst.21.3.1362 12523697

[81] Hagenaars TJ Dekker A de Jong MC Eble PL. Estimation of foot and mouth disease transmission parameters, using outbreak data and transmission experiments. Rev Sci Tech (2011) 30(2):467–81. 21961219

[82] Sørensen JH Mackay DKJ Jensen C Donaldson AI. An integrated model to predict the atmospheric spread of foot-and-mouth disease virus. Epidemiol Infect (2000) 124:577–90. 10.1017/S095026889900401X 10982082 PMC2810944

[83] Mikkelsen T Alexandersen S Astrup P Champion HJ Donaldson AI Dunkerley FN Investigation of airborne foot-and-mouth disease virus transmission during low-wind conditions in the early phase of the UK 2001 epidemic. Atmos Chem Phys (2003) 3(6):2101–10. 10.5194/acp-3-2101-2003

[84] Hess GD Garner MG Yang XA Sensitivity analysis of an integrated modelling approach to assess the risk of wind-borne spread of foot-and-mouth disease virus from infected premises. Environ Model Assess (2008) 13:209–20. 10.1007/s10666-007-9097-3

[85] Donaldson AI Sellers RF Lacey J Quantitative data on airborne foot-and-mouth disease virus: its production, carriage and deposition [and discussion]. Philos Trans R Soc B Biol Sci (1983) 302(1111):529–34. 10.1098/rstb.1983.0072

[86] Weesendorp E Stegeman A Loeffen WLA. Dynamics of virus excretion via different routes in pigs experimentally infected with classical swine fever virus strains of high, moderate or low virulence. Vet Microbiol (2009) 133(1–2):9–22. 10.1016/j.vetmic.2008.06.008 18635323

[87] Thomson GR Vosloo W Bastos ADS Foot and mouth disease in wildlife. Virus Res (2003) 91:145–61. 10.1016/S0168-1702(02)00263-0 12527441

[88] Alexandersen S Brotherhood I Donaldson AI. Natural aerosol transmission of foot-and-mouth disease virus to pigs: minimal infectious dose for strain O1 Lausanne. Epidemiol Infect (2002) 128:301–12. 10.1017/S0950268801006501 12002549 PMC2869824

[89] Coleman M Marks H. Topics in dose-response modeling. J Food Prot (1998) 61(11):1550–9. 9829203 10.4315/0362-028x-61.11.1550

[90] Buchanan RL Smith JL Long W. Microbial risk assessment: dose-response relations and risk characterization. Int J Food Microbiol (2000) 58(3):159–72. 10.1016/S0168-1605(00)00270-1 10939266

[91] Hermann J Hoff S Munoz-Zanzi C Yoon KJ Roof A Burkhardt A Effect of temperature and relative humidity on the stability of infectious porcine reproductive and respiratory syndrome virus in aerosols. Vet Res (2007) 38:81–93. 10.1051/vetres:2006044 17156739

[92] Lowen AC Mubareka S Steel J Palese P. Influenza virus transmission is dependent on relative humidity and temperature. PLoS Pathog (2007) 3(10):e151. 10.1371/journal.ppat.0030151 17953482 PMC2034399

[93] Steel J Palese P Lowen AC. Transmission of a 2009 pandemic influenza virus shows a sensitivity to temperature and humidity similar to that of an H3N2 seasonal strain. J Virol (2011) 85(3):1400–2. 10.1128/JVI.02186-10 21084485 PMC3020521

[94] Mbithi JM Springthorpe VS Sattar SA. Effect of relative humidity and air temperature on survival of hepatitis A virus on environmental surfaces. Appl Environ Microbiol (1991) 57(5):1394–9. 1649579 10.1128/aem.57.5.1394-1399.1991 PMC182960

