# Supplementary material for: Relevance of Indirect Transmission for Wildlife Disease Surveillance
Source: Front Vet Sci. 2016 Nov 30;3:110. doi: 10.3389/fvets.2016.00110 (PMC5127825; doi:10.3389/fvets.2016.00110)
Supplement: Supplementary file 1 [file Data_Sheet_1.PDF]

Relevance of indirect transmission for wildlife disease surveillance.

Supplementary Material:

ODD Model description

Martin Lange[1], Stephanie Kramer-Schadt[2], Hans-Hermann Thulke[1]

[1]Department of Ecological Modelling, Helmholtz Centre for Environmental Research Leipzig - UFZ, Leipzig, Germany

[2]Leibniz Institute for Zoo and Wildlife Research, Alfred-Kowalke-Str. 17, Berlin D-10315, Germany

ODD protocol

Model description

Overview

The FMD wildlife model is a compilation of a spatially explicit, stochastic, individual based demographic model for wild boars (*Sus scrofa*) in a geographic area with suitable habitat. Superimposed is an infection and disease course model for the FMDV. The model is documented following the ODD protocol (Overview, Design, Details [1,2]).

Purpose

The aim of the modelling study was to provide an experimental environment to test the hypothesis that neglect of pathogen persistence outside its host is an inappropriate simplification from the perspective of surveillance or contingency planning. The model was designed to compare the predictions between explicit indirect transmission (IT) and equivalently parameterised direct transmission (DT).

State variables and scales

The model comprises two major components: spatial habitat units and wild boar individuals. All processes take place on a raster map of spatial habitat units. Each cell represents a functional classification of a landscape denoting habitat quality and a scalar value denoting environmental pathogen load. The cells of the model landscape represent 4 km² (2 × 2 km), encompassing a boar group's core home range [3]. State variables comprise habitat quality of the grid cells for boar. At run time, habitat quality is interpreted as breeding capacity, i.e. the number of female boars that may to have offspring (explicit density regulation; [4]). The habitat cell account for FMDV through a state-variable representing environmental contamination and accumulates infection pressure as shed by viraemic animals.

State variables of host individuals are the wild boar's age in weeks (where one week represents the approximate FMD infectious period in wild boar; [5,6]), resulting in age-classes: piglet (< 8 months ± 6 weeks), sub-adult (< 2 years ± 6 weeks) and adult [7]. Each host individual has a location, which denotes its home range cell on the raster grid as well as its family group. The individual host animal comprises an epidemiological status (*susceptible, infected* or *immune* after recovery or due to transient maternal antibodies). Sub-adult wild boar may disperse during the dispersal period (i.e. early summer).

Process overview and scheduling

The model proceeds in weekly time steps, and weekly processes were performed as follows (see Figure S1): virus release, infection, dispersal of subadults, reproduction, death and ageing, executed in the given order. In the first week of each year, mortality probabilities are assigned stochastically to represent annual fluctuations in boar living conditions, and boars are assigned to breed or not, according to the carrying capacity of their home range cell.

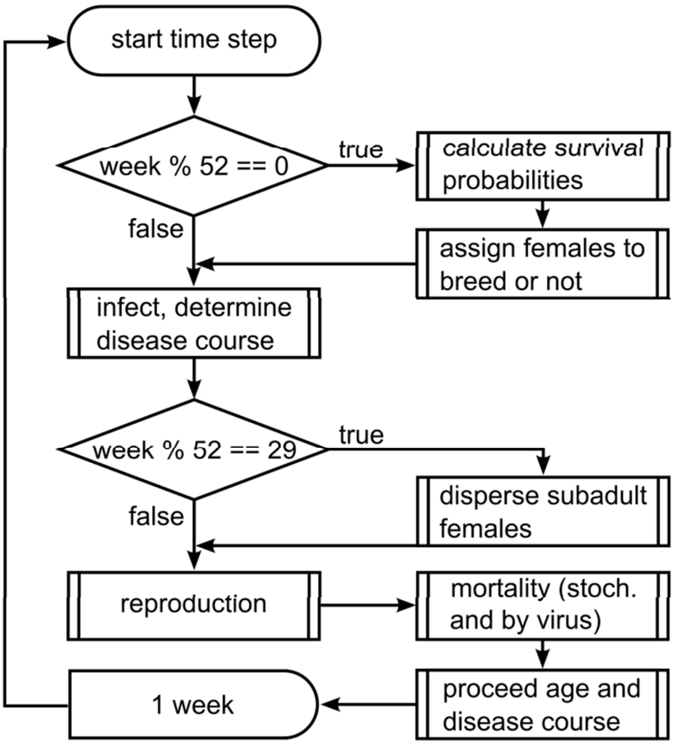

Figure S1: Flow chart of the scheduling of sub-models.

Design concepts

Wild boar population dynamics emerge from individual behaviour, defined by age-dependent seasonal reproduction and mortality probabilities and age- and density-dependent dispersal behaviour, all including stochasticity. The epidemic course in the DT model emerges from virus transmission within and between groups and wild boar dispersal. The epidemic course in the IT model emerges from virus excretion by infectious hosts, survival dynamics of infectious virus outside the host (e.g. [8]), contact to infectious doses and wild boar dispersal.

We included stochasticity by representing demographic, behavioural and pathogen parameters as probabilities or probability distributions. Annual fluctuations of living conditions are realised by annually varying mortality rates.

Details

Initialisation

The model landscape represents 60 km × 60 km of connected wildlife habitat without barriers. The specified extent ensures that the epidemic wave does not reach the edge of the landscape before detection in any simulation. The 900 grid cells were randomly initialised with integer values of local breeding capacity in range 0...3. Breeding capacity was scaled to result in an average wild boar density of 5 hosts per km² in January, i.e. before the reproductive season. The average population size in January was 18,000 individuals.

One boar group was released to each habitat cell, where group size is six times breeding capacity. Initial age distributions were taken from the results of a 100 years model run (see Table S1) [9].

Table S1: Initial age distributions of boar [9] and deer.

Upper age bound (years)	1	2	3	4	5	6	7	8	9	10	11
Proportion	0.38	0.24	0.15	0.09	0.06	0.03	0.02	0.01	0.01	0.01	0.00

Input

The applied model setup does not include any external inputs or driving variables.

Submodels

Submodels are described in the order of their execution. Parameters and their values are listed in Table S2 in section "Parameters".

Virus release

The virus was released to the population by infection of five wild boars, randomly selected from the nine most central habitat cells. Release takes place in the first week of the sixth year of simulation (see 'Simulation experiments') to allow population dynamics to be established. Introduction was chosen in the season of most likely establishment of the infection according to the increasing population numbers, i.e. at the start of the reproductive season of wild boar.

Disease course

The disease course following infection is modelled for each infected individual. The infectious period of a host t_{inf} is one week. After the infectious period, hosts achieve lifelong immunity. We assumed minimum case lethality [5,6].

If the individual has maternal antibodies it is immune if its age T_k is below the number of weeks of immunisation due to maternal antibodies T_{immune}.

Virus transmission

Direct transmission (DT)

Transmission is modelled stochastically. Parameters determine the probability of contracting the infection from an infectious group mate $P_{\text{inf}}^{(i)}$ and the probability of contracting the infection from an infectious animal in a neighbouring group $P_{\text{inf}}^{(e)}$ (3 × 3 neighbourhood) during one week. For each susceptible animal the probability of becoming infected accumulates over all infectious animals within the group and in the neighbourhood:

$$\Pi_i = 1 - (1 - P_{\text{inf}}^{(i)})^{I_i} \cdot (1 - P_{\text{inf}}^{(3)})^{\Sigma_j I_j} \qquad (1)$$

where λ_i is the number of infected individuals in the group i and λ_j is the number of infected individuals in groups of the 8 neighbouring cells j. The model iterates over all individuals and stochastically sets each susceptible individual to infected if a uniformly distributed random number r drawn from $U(0, 1)$ is smaller than Π_i of its home cell.

Indirect transmission (IT)

We modelled indirect virus transmission via excretion of infectious material, decay of infectious material by time in the environment (i.e. outside of host individuals) and contact of hosts to infectious material in the environment. At contact, we modelled the effective infection stochastically with the event probability derived from a standard dose-response relation.

The weekly dynamics of the pathogen pool used in the model are based on parameters available from literature on a daily basis. Temporal evolution of the pathogen pool C of each cell is an exponential decay process and the term of pathogen load added to the cell:

$$\frac{dC}{dt} = -\lambda C + s \qquad (2)$$

with λ being the decay constant $\lambda = \ln(2)/T_{1/2}$, *s* being the pathogen added to the cell per time unit and *t* being time in weeks. Solve:

$$C(t) = \left(C_0 - \frac{s}{\lambda}\right) \cdot e^{-\lambda t} + \frac{s}{\lambda} \quad (3)$$

Within one time step, *s* is constant. Thus, the pathogen pool can be calculated analytically as:

$$C_{t+1} = \left(C_t - \frac{s}{\lambda}\right) \cdot e^{-\lambda} + \frac{s}{\lambda} \quad (4)$$

The available dose for uptake during the time step is

$$\overline{C} = \int_t^{t+1} C(t)dt = \frac{C_t \cdot (1 - e^{-\lambda}) + s}{\lambda} + \frac{s \cdot (e^{-\lambda} - 1)}{\lambda^2} \quad (5)$$

The source term for a cell is determined from the number of infectious hosts in the cell and in neighbouring cells. Hosts in infectious state excrete infectious material with constant daily rate (parameter *g*), measured in $TCID_{50}$ per day. A host animal spends a portion of daytime (parameter p_t) in contact areas, i.e. areas subsequently reached by neighbouring animal groups. Accordingly, excreted infectious material is distributed to different cells: $g(1 - p_t)$ doses adding to the pool of the home cell of the host, while $1/8\, g\, p_t$ doses are added to each neighbouring cell. Therefore, the source term of a cell on a weekly basis is:

$$s = 7g \cdot \left((1 - p_t) I_i + 1/8\, p_t \sum_j I_j\right) \quad (6)$$

Per host, individual contact to infectious material in the environment is determined as constant (small) share (parameter *u* on a daily basis) of the available dose $\overline{C}_{ij}$ in its home range cell. The weekly contact dose *CD* is

$$CD = 7u\overline{C} \quad (7)$$

Effective infection after contact to a particular dose of infectious material is modelled stochastically as a binomial chance process so that the individual's weekly probability of becoming infected follows an exponential dose-response relation:

$$P_{CD} = 1 - (1 - P_{TCID50})^{CD} \quad (8)$$

with P_{TCID50} being the probability of infection after contact to one $TCID_{50}$ dose. Figure S2 shows the dose response curve for $P_{TCID50} = 0.003$ [10,11].

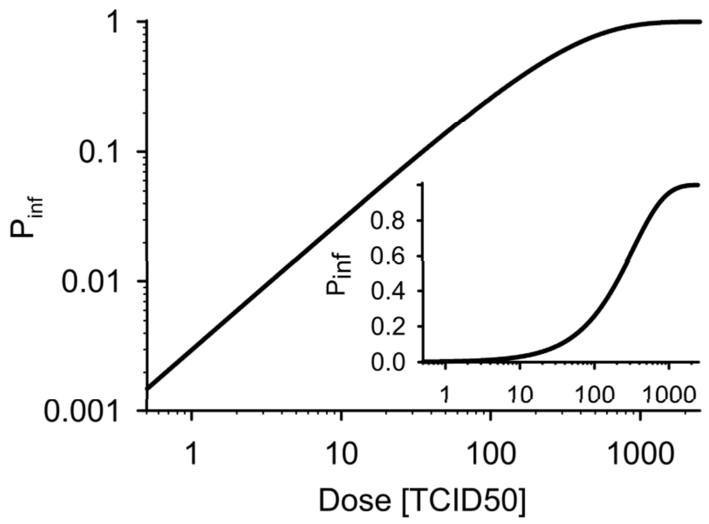

Figure S2: Dose-response curves for wild boar (P_{TCID50} =0.003). Inset: linear ordinate.

Group splitting

Groups split in specified weeks of the year only. The model collects subadult females without offspring able to move in each of the *N* habitat cells.. Then, all groups to split are extracted, matching the conditions of containing a number of females exceeding the cell breeding capacity and containing at least a specified number of subadults to move N_{disp}.

Splittable groups are iterated randomly. For each of them, an empty habitat cell (breeding capacity above 0) within Euclidean distance D_{disp} is selected randomly, excluding the source cell. All moving individuals out of the considered source group found a new group on that cell. If no empty habitat is available, disperser females cannot move.

Reproduction

Females reproduce only once a year if they are at least in the subadult age class. Individual females reproduce depending on the season with a peak in March (see [12], Figure S3A).

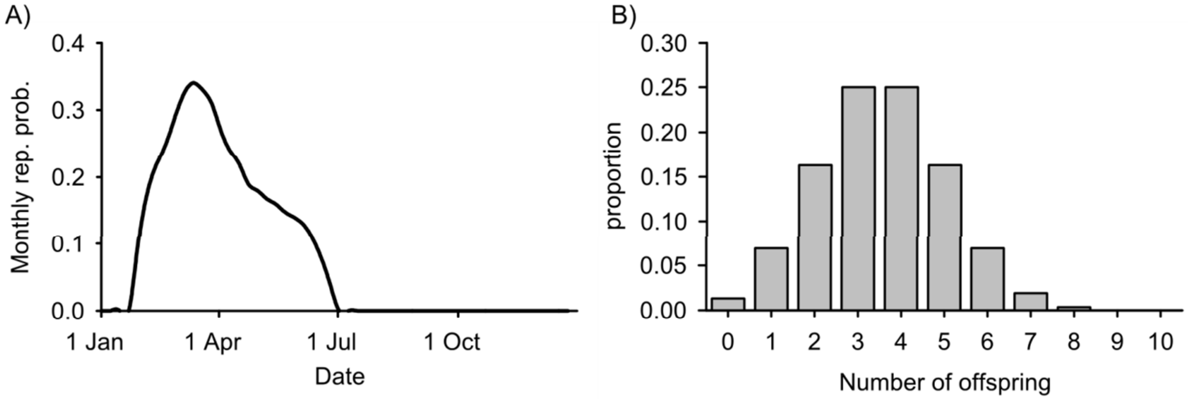

Figure S3: A) Monthly reproduction probabilities for wild boar. B) Breed count distributions for wild boar [12, Appendix C,13].

In the first week of the year, we check whether female individuals are are able to breed. All boar females not exceeding their habitat cells' breeding capacity CC_{ij}, starting with the oldest individuals, are allowed to breed.

The week of the year to breed is assigned according to weekly reproduction probabilities, derived from monthly probabilities and the number of weeks in the month (Figure S3a).

Litter size is drawn from a pre-specified truncated normal distribution (Figure S3b). Litter size is reduced to a constant fraction for infected individuals. Litter size of transient shedders and lethally infected hosts is multiplied with the reduction factor α_f.

Depending on the disease state of the breeding individual, its piglet's disease states are adjusted. Susceptible individuals and transient non-shedders produce susceptible offspring, immune individuals produce immune offspring with maternal antibodies. Transient shedders and lethally infected individuals yield offspring, each one lethally infected with a given probability of prenatal infection P_{PI}.

Death

Iterating over the entire population, each individual either dies stochastically due to age class dependent mortality probabilities by drawing a random number or due to reaching a certain maximum age.

Stochastic baseline mortality is age-dependent and adjusted to annual survival estimates found in the literature. These survival estimates together with the reported variability (see Table S2) determine the Gaussian distributions we draw from the random survival in the model on a yearly basis (SP_{Year}). The stochastic effect resembles 'good' or 'bad' years for the host species, i.e. environmental noise. In the model application the Gaussian distributions are

cut symmetrically around the mean. Per time step, we apply the adjusted age-dependent mortality (PM_{Week}) to the individual:

$$PM_{Week} = 1 - (SP_{Year})^{1/52}$$

Ageing

The ageing process iterates over all individuals. For each individual k, age T_k is incremented by one week and disease state transitions are performed. Transient shedders are converted to immune after their individual infectious period t_{inf}.

An individual k protected by maternal antibodies turns susceptible if reaching an age T_k of the protection time T_{immune}. Maternal antibodies themselves vanish on reaching a maximum age of maternal antibody persistence T_{anti}.

After disease state transition the age of the infection is incremented by one week if the individual is not susceptible.

Parameters, simulation experiments, analysis

Parameters

Model parameters of the indirect transmission model are shown in Table S2.

Table S2: Model parameters. Default values in bold.

Symbol	Description	Value	Source / details
α_f	Fertility reduction if ill	0.625	
P_{PI}	Probability of prenatal infection	0.5	
$T_{\max}$	Maximum age of boar / deer	11 years (572 weeks)	[14]
T_{anti}	Maximum persistence of maternal antibodies	15 weeks	[15]
T_{immune}	Maximum duration of immunity by maternal antibodies	12 weeks	[15]
$P_{\inf}^{(i)}$	Infection probability by direct transmission within wild boar groups	0.0 (IT); 0.087 (DT)	DT scaled to resemble IT
$P_{\inf}^{(e)}$	Infection probability by direct transmission between groups	0.0 (IT); 0.0038 (DT)	
g	Daily excretion per boar	10^6 $TCID_{50}$	[11,16,17]
u	Ingestion factor	(32, 16, 8, …, 1/8, 1/16)×10^{-6} (IT); 0 (DT)	Scaled to $u = 4/(g \times T_{1/2})$
P_{TCID50}	Probability of infection resulting from ingestion of one $TCID_{50}$ dose	0.003	[10,17]
$T_{1/2}$	Half-life time of environmental	1/8, 1/4, 1/2, …,	[8,18,19]

Symbol	Description	Value	Source / details
	contamination	32, 64 (IT); 0 (DT)	
p_t	Neighbourhood forage proportion	0.25	
D_{disp}	Maximum dispersal distance for subadult females	6 km	[20]
N_{disp}	Minimum number of subadult females for dispersal	2	
$SP^{(a)}_{mean}$ / $SP^{(a)}_{\min}$	Mean / minimum annual survival rate	0.65 / 0.4	[21]
$SP^{(y)}_{mean}$ / $SP^{(y)}_{\min}$	Mean / minimum annual survival rate	0.65 / 0.4	[22]
$SP^{(p)}_{mean}$ / $SP^{(p)}_{\min}$	Mean / minimum annual survival rate	0.5 / 0.1	[21]

Parameterisation of transmission

In the DT model the transmission is defined by scaling the two parameters $P^{(i)}_{\inf}$ and $P^{(e)}_{\inf}$. In the IT model, an analogue to $P_{\inf}$ can be calculated from Equation (8) and the dose available from one infectious host. To calculate the available dose, Equation (5) is applied for one week after infection (i.e. parameter infectious period) including the excretion into the environment (i.e. s > 0) and for infinite time without further excretion. The total available dose over time is

$$\bar{C}^{\infty} = \int_0^1 C^+(t)\,dt + \int_0^{\infty} C^-(t)\,dt \qquad (9)$$

Where $C^+(t)$ is the pathogen pool with pathogen excretion starting with $C_0 = 0$ (Equation 3). $C^-(t)$ is the pathogen pool without pathogen excretion for an initial pool equal to the value after the first week (i.e. $C_0 = C^+(1)$). Solve:

$$\bar{C}^{\infty} = \frac{s}{\lambda} \qquad (10)$$

Or, without stressing mathematics, it is the product of added material s and average lifetime of the pathogen in the environment $\tau = 1/\lambda$.

With Equations (7) and (8) this gives

$$P^{(i)*}_{\inf} = 1-(1-P_{TCID50})^{7us_i/\lambda} \qquad (11)$$

$$P^{(e)*}_{\inf} = 1-(1-P_{TCID50})^{7us_e/\lambda} \qquad (12)$$

with newly added pathogen $s_i = 7g \cdot (1 - p_t)$ for within-group transmission and $s_e = 7g \cdot 1/8\, p_t$ for between-group transmission.

By choosing $P_{\text{inf}}^{(i)} = P_{\text{inf}}^{(\text{i})*}$ and $P_{\text{inf}}^{(e)} = P_{\text{inf}}^{(e)*}$, both models produce the same basic reproductive number R_0 (for validation see **Fehler! Verweisquelle konnte nicht gefunden werden.**).

Parallel of R_0 in DT and IT model

The DT model was parameterised to mimic the IT model in terms of the basic reproduction number R_0. Accounting for transmission within and between groups, R_0 was calculated for both scales of spatial transmission separately. This gives the expected number of infections from one case to its group-mates $R_0^{(i)}$ and to the animals of neighbouring groups $R_0^{(\text{e})}$, summing up to $R_0 = R_0^{(i)} + R_0^{(\text{e})}$.

In the DT model with an infectious period of one week, R_0 is a linear function of P_{inf}:

$$R_0^{(i)} = S_i P_{\text{inf}}^{(i)} \tag{13}$$

$$R_0^{(\text{e})} = S_e P_{\text{inf}}^{(\text{e})} \tag{14}$$

S_i is the number of susceptible hosts in the group of the infectious individual. S_e is the number of susceptible hosts in its neighbouring groups.

We can calculate R_0 from the parameters of the IT model using Equations (11) and (13) for within-group transmission and Equations (12) and (14) for between-group transmission:

$$R_0^{(i)} = S_i \cdot \left(1 - (1 - P_{TCID50})^{7us_i/\lambda}\right) \tag{15}$$

$$R_0^{(\text{e})} = S_e \cdot \left(1 - (1 - P_{TCID50})^{7us_e/\lambda}\right) \tag{16}$$

The exponent in Equations (15) and (16) can be transformed to $7us/\lambda = 7usT_{1/2} / \ln(2)$. Thus, R_0 in the IT model can be kept constant over arbitrary pathogen half-life $T_{1/2}$ by compensatory scaling of the uptake u, i.e. $u \cdot T_{1/2}$ is constant (see Figure 4). With pathogen half-life approaching zero, the IT model becomes equivalent to the DT model as pathogen uptake becomes instantaneous.

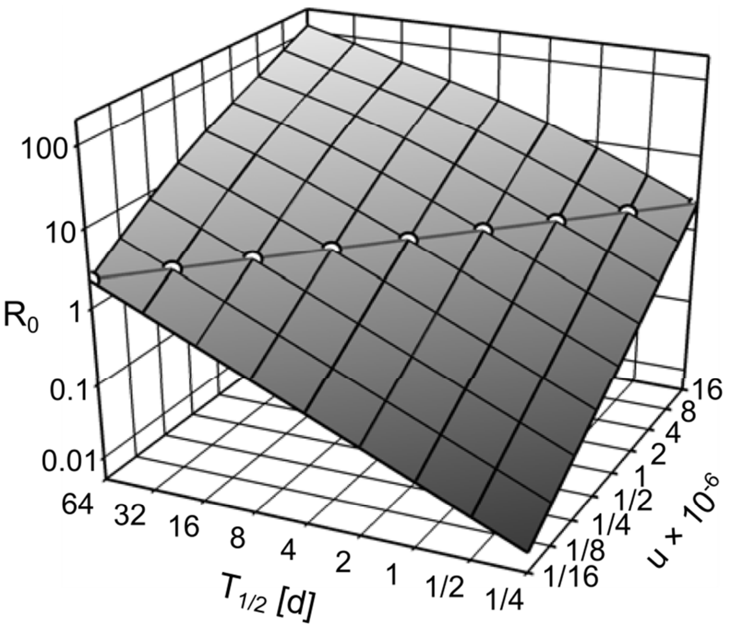

Figure 4: R_0, depending on the environmental pathogen half-life $T_{1/2}$ and on the uptake rate u. Circles along the diagonal line show the realisations of $T_{1/2}$ and u used in the simulation experiments ($u \cdot T_{1/2} = 4 \cdot 10^{-6}$), resulting in $R_0 = 2.259$.

Independent variables

The primary independent variable was the pathogen half-life $T_{1/2}$.

Simulation experiments

We performed simulations for the IT model with environmental pathogen half-life $T_{1/2} \in \{1/8, 1/4, 1/2, ..., 32, 64\}$ days (Figure 4). To keep R_0 constant over all IT simulations, we scaled u according to $u = 4 \cdot 10^{-6} / T_{1/2}$. All parameter combinations resulted in $R_0 = 2.259$. For comparison, we repeated the simulations with the DT model. To achieve the same R_0 as the IT model, transmission parameters were scaled to $P_{\text{inf}}^{(i)} = 0.087$, $P_{\text{inf}}^{(e)} = 0.00379$. Each parameter set was repeated 500 times.

We performed supplementary simulations to measure an individual-based equivalent of R_0 [23] in order to verify accordance of the transmission model with the theoretical calculations for the basic reproduction number. This was achieved by allowing only the first disease case per model run to be infectious and count of the number of secondary infection in the initially infected cell and in its neighbouring cells. The theoretical calculations neglect population turnover, therefore in a third set of simulations reproduction and mortality were deactivated from the week of pathogen introduction onwards. The model runs for 100 times

the pathogen half-life after the initial infection to make sure that the environmental reservoir completely decayed and no secondary infections were missed in the analysis.

Dependent variables

We recorded sero-prevalence time series for each run on a weekly basis as a first order dependent variable. These prevalence time series were then used to determine second-order dependent variables: (1) time to detection for fixed weekly sample sizes, (2) size of the outbreak at the time of detection and, (3) sample sizes needed to detect the disease within an *a priori* specified time frame. For second-order dependent variables see section 'Analysis'.

Analysis

We mimicked systematic surveillance on the sero-prevalence outcome p of the DT and the IT model deriving the following second-order dependent variables from prevalence time series.

Time to detection

Given a weekly sample size n and sero-prevalence p, the probability to not find any sero-positives in a particular week t is

$$\hat{P}_0(t) = (1 - p(t))^n \tag{17}$$

The probability of not finding any sero-positives until the given week can be determined as:

$$P_0(t) = \prod_{i=0}^{t} \hat{P}_0(t) \tag{18}$$

Hence, the probability to detect the disease until the given week is

$$P_D(t) = 1 - \prod_{i=0}^{t} \hat{P}_0(t) \tag{19}$$

For each model run, the first week of $P_D(t) \geq 0.95$ determines the time of detection. Subtracting the week of virus incursion, this gives the time to detection t_D of the individual run. The geometric mean of the distribution over the runs gives the time to detection t_D with 95% confidence.

Sample sizes for the underlying surveillance scheme were determined on a monthly basis according to the following equation [24]:

$$n_{month} = \left(1-(1-CL)^{\frac{1}{N\cdot p}}\right)\cdot\left(N-\frac{N\cdot p-1}{2}\right) \quad (20)$$

with true population size *N*. Parameters of interest were $CL = 95\%$, $p = 5\%$ and 1%. The required sample size was 58.3 per month (14 per week) for $p = 5\%$ and 295.6 per month (69 per week) for $p = 1\%$.

Outbreak size

The area affected by the disease (area of cells infected) before detection A_{aff} was determined as a measure of the spatial extent of the outbreak.

Required sample size

The probability to detect the disease before the given week is calculated according to equation (19). This gives the weekly sample size needed to detect the disease in a given time frame *t* for a given sero-prevalence time series:

$$n_D = \frac{\ln(1-\mathrm{CL})}{\ln\prod_{i=0}^{t}(1-\mathrm{p(i)})} \quad (21)$$

We calculated the required weekly sample sizes for each model run.

Statistical analysis

For each simulated value of $T_{1/2}$ in the IT model, we compared distributions of time to detection t_D and weekly sample size needed n_D to the outcome of the DT model using the Mann–Whitney U test (H_0: distribution with IT not greater than distribution with DT). Similarly, distributions of A_{aff} were compared to the outcome of the DT model using the Mann–Whitney U test (H_0: distribution with IT not less than distribution with DT). Significance was defined as p-value < 0.01.

References

1. Grimm V, Berger U, Bastiansen F, Eliassen S, Ginot V, et al. (2006) A standard protocol for describing individual-based and agent-based models. Ecological Modelling 192: 115-126.
2. Grimm V, Berger U, DeAngelis DL, Polhill JG, Giske J, et al. (2010) The ODD protocol: A review and first update. Ecological Modelling 221: 2760-2768.

3. Leaper R, Massei G, Gorman ML, Aspinall R (1999) The feasibility of reintroducing Wild Boar (Sus scrofa) to Scotland. Mammal Review 29: 239-258.
4. Jedrzejewska B, Jedrzejewski W, Bunevich AN, Milkowski L, Krasinski ZA (1997) Factors shaping population densities and increase rates of ungulates in Bialowieza Primeval Forest (Poland and Belarus) in the 19th and 20th centuries. Acta Theriologica 42: 399-451.
5. Mohamed F, Swafford S, Petrowski H, Bracht A, Schmit B, et al. (2011) Foot-and-Mouth Disease in Feral Swine: Susceptibility and Transmission. Transboundary and Emerging Diseases 58: 358-371.
6. Breithaupt A, Depner K, Haas B, Axandrov T, Polihronova L, et al. (2012) Experimental infection of wild boar and domestic pigs with a Foot and mouth disease virus strain detected in the southeast of Bulgaria at the end of 2010. Veterinary Microbiology 159: 33-39.
7. Keuling O, Stier N, Roth M (2008) Annual and seasonal space use of different age classes of female wild boar *Sus scrofa* L. European Journal of Wildlife Research 54: 403-412.
8. Bøtner A, Belsham GJ (2012) Virus survival in slurry: Analysis of the stability of foot-and-mouth disease, classical swine fever, bovine viral diarrhoea and swine influenza viruses. Veterinary Microbiology 157: 41-49.
9. Kramer-Schadt S, Fernández N, Eisinger D, Grimm V, Thulke HH (2009) Individual variations in infectiousness explain long-term disease persistence in wildlife populations. Oikos 118: 199-208.
10. French NP, Kelly L, Jones R, Clancy D (2002) Dose-Response Relationships for Foot and Mouth Disease in Cattle and Sheep. Epidemiology and Infection 128: 325-332.
11. Garner MG, Hess GD, Yang X (2006) An integrated modelling approach to assess the risk of wind-borne spread of foot-and-mouth disease virus from infected premises. Environmental Modeling and Assessment 11: 195-207.
12. EFSA (2012) Scientific Opinion on foot and mouth disease in Thrace. The EFSA Journal 10: 2635.
13. Bieber C, Ruf T (2005) Population dynamics in wild boar *Sus scrofa*: ecology, elasticity of growth rate and implications for the management of pulsed resource consumers. Journal of Applied Ecology 42: 1203-1213.
14. Jezierski W (1977) Longevity and mortality rate in a population of wild boar. Acta Theriologica 22: 337-348.

15. Depner K, Müller T, Lange E, Staubach C, Teuffert J (2000) Transient classical swine fever virus infection in wild boar piglets partially protected by maternal antibodies. Deutsche Tierärztliche Wochenschrift 107: 66-68.
16. Sørensen JH, Mackay DKJ, Jensen C, Donaldson AI (2000) An Integrated Model to Predict the Atmospheric Spread of Foot-and-Mouth Disease Virus. Epidemiology and Infection 124: 577-590.
17. Hess GD, Garner MG, Yang X (2008) A Sensitivity Analysis of an Integrated modelling Approach to Assess the Risk of Wind-borne Spread of Foot-and-mouth Disease Virus from Infected Premises. Environmental Modeling and Assessment 13: 209-220.
18. Turkish State Meteorological S (2012) Turkish State Meteorological Service.
19. Deutscher W (2012) Deutscher Wetterdienst.
20. Sodeikat G, Pohlmeyer K (2003) Escape movements of family groups of wild boar Sus scrofa influenced by drive hunts in Lower Saxony, Germany. Wildlife Biology 9: 43-49.
21. Focardi S, Toso S, Pecchioli E (1996) The population modelling of fallow deer and wild boar in a Mediterranean ecosystem. Forest Ecology and Management 88: 7-14.
22. Gaillard JM, Vassant J, Klein F (1987) Some characteristics of the population dynamics of wild boar (Sus scrofa scrofa) in a hunted environment. Gibier Faune Sauvage 4: 31-47.
23. Almberg ES, Cross PC, Johnson CJ, Helsey DM, Richards BJ (2011) Modeling Routes of Chronic Wasting Disease Transmission: Environmental Prion Persistence Promotes Deer Population Decline and Extinction. PLoS ONE 6: e19896.
24. Cannon RM (2001) Sense and sensitivity - designing surveys based on an imperfect test. Preventive Veterinary Medicine 49: 141-163.
